# Descriptions of two new species of *Platygaster* Latreille that attack gall midges (Diptera, Cecidomyiidae) with notes on their biology (Hymenoptera, Platygastridae)

**DOI:** 10.3897/zookeys.754.23296

**Published:** 2018-05-03

**Authors:** Kazunori Matsuo, Tomohisa Fujii, Makoto Tokuda, Tomoko Ganaha-Kikumura, Junichi Yukawa, Kenzou Yamagishi

**Affiliations:** 1 Biosystematics Laboratory, Faculty of Social and Cultural Studies, Kyushu University, Fukuoka812–0395, Japan; 2 NARO Kyushu Okinawa Agricultural Research Center, Kumamoto 861–1192, Japan; 3 Laboratory of Systems Ecology, Faculty of Agriculture, Saga University, Saga 840–8502, Japan; 4 Okinawa Prefectural Agricultural Research Center, Okinawa 901–0336, Japan; 5 Entomological Laboratory, Faculty of Agriculture, Kyushu University, Fukuoka 812–8581, Japan; 6 Entomological Laboratory, Faculty of Agriculture, Meijo University, Nagoya 468–8502, Japan

**Keywords:** *Platygaster ingeniosus*, *Platygaster urniphila*, platygastrids, taxonomy

## Abstract

*Platygaster ingeniosus* Matsuo & Yamagishi, **sp. n.** and *P. urniphila* Matsuo & Yamagishi, **sp. n.** (Hymenoptera: Platygastridae) are described from Japan. The former species is an egg-larval solitary parasitoid of *Masakimyia pustulae* Yukawa and Sunose (Diptera: Cecidomyiidae). The latter species is an egg-larval gregarious parasitoid of *Rhopalomyia longitubifex* (Shinji) (Diptera: Cecidomyiidae).

## Introduction

The genus *Platygaster* Latreille (Hymenoptera: Platygastridae) contains 601 species, of which approximately 300 species have been described from various biogeographic regions during the last two decades (e.g., Buhl 2004a, b, 2006a, b). Most *Platygaster* species share the following morphological characters: antenna with 10 antennomeres; scuto-scutellar suture deep and usually forming a fovea; mesoscutellum rounded, usually without a distinct spine or tuft of hairs; fore and hind wings without venation; T1 without dense hairs. In addition, *Platygaster* includes some exceptional species that do not share all of the aforementioned characters (Buhl 1994a, b, 2001a, b, 2003a, b).

Today, nine species of *Platygaster* are known to occur in Japan, of which seven parasitize gall midge species (Diptera: Cecidomyiidae) (Ashmead 1904; Ishii 1953; Yoshida and Hirashima 1979; Yamagishi 1980; Buhl and Duso 2008; Vlug 1995). In addition to the nine known Japanese species, two unidentified species of *Platygaster* have been recognized to parasitize gall midge larvae (Sunose 1984; Ganaha et al. 2007). One species is an egg-larval parasitoid of *Masakimyia pustulae* Yukawa and Sunose (Diptera: Cecidomyiidae) that induces leaf galls on *Euonymus japonicus* (Celastraceae) (Yukawa and Sunose 1976; Sunose 1984). This *Platygaster* species avoids hyperparasitism by manipulating larvae of the host gall midge to make leaf galls thicker (Fujii et al. 2014). Another species parasitizes *Rhopalomyia longitubifex* (Shinji) (Diptera: Cecidomyiidae) that induces axillary bud galls on Artemisia indica var. maximowiczii (Asteraceae) (Yukawa and Masuda 1996; Ganaha et al. 2007).

As demonstrated by Askew (1975) for parasitoids of Cynipidae (Hymenoptera), Yukawa et al. (1981) also divided parasitoids of gall-inducing cecidomyiids (Diptera: Cecidomyiidae) into two groups, early and late attackers, according to their parasitic strategies. Early attackers (koinobionts) are host-specific endoparasitoids that oviposit into host eggs or younger host larvae before galls start to develop (Sunose 1984, 1985a; Tabuchi and Amano 2004). In contrast, late attackers (idiobionts) are polyphagous ectoparasitoids attacking final (third) instars or pupae. Species of *Platygaster* that are associated with gall-inducing cecidomyiids are known as typical early attackers (Askew 1975). Host specificity has been paid special attention in behavioral and ecological studies of *Platygaster*, particularly host–parasitoid interactions. For example, Stireman III et al. (2006) demonstrated host-associated genetic differentiation in *Platygaster variabilis* Fouts that attacks *Rhopalomyia solidaginis* (Loew) (Diptera: Cecidomyiidae). Yamagishi (1980) reported that larvae of *Rabdophaga rosaeformis* Kovalev (Diptera: Cecidomyiidae) parasitized by *Platygaster stimulator* Yamagishi mature in summer whereas unparasitized *R. rosaeformis* larvae pass through the summer as first instars. In terms of reproductive strategy, some species including *P. robiniae* Buhl and Duso are known to be gregarious parasitoids (Kim et al. 2011). In addition, polyembryony, the production of genetically identical embryos from a single egg through clonal division, has been found in several species of *Platygaster* such as *P. feltii* Fouts, and *P. vernalis* (Myers) (Leiby and Hill 1924; Segoli et al. 2010).

In order to contribute to further taxonomic and ecological studies of platygastrid parasitoids, we intend in this paper to identify the two undescribed species of *Platygaster* and to provide information on their host range and reproductive strategies.

## Materials and methods

Galls of *Masakimyia pustulae* and *Rhopalomyia longitubifex* were collected from Kyushu, Japan, in 2007–2017 to rear sufficient numbers of adults of *Platygaster* species for taxonomic study. In rearing *Platygaster* species that attacks *Rhopalomyia longitubifex*, the number of males and females emerged from one host larva were recorded to confirm its gregarious parasitism.

For morphological observation, adult parasitoids were preserved in 70–75% ethanol and subsequently dried from ethanol using the method described in Matsuo and Yukawa (2009). Specimens were observed under a binocular microscope (LEICA S8APO). Several specimens were gold-coated for microphotography with a JEOL JSM-5600LV scanning electronic microscope. High-resolution bright field images were taken with LEICA S8APO and CANON EOS D600 (Matsuo et al. 2012).

To compare morphological characters between known and the two Japanese species, we referred to original descriptions, redescriptions or keys for 512 (85.2%) out of 601 known species (Suppl. material 1). Unfortunately, we could not obtain adequate morphological information on the remaining 89 species. In addition to literature survey, we examined high-resolution images of the type specimen of a Japanese species, *Platygaster gifuensis* (Ashmead) that has been kept in the Smithsonian National Museum of Natural History, Washington, DC, USA. Adult morphological terminology follows Masner and Huggert (1989), except for head and mesosoma, which follows Mikó et al. (2007).

Holotypes and paratypes of the new species are deposited in the collection of the Biosystematics Laboratory, Faculty of Social and Cultural Studies, Kyushu University, Fukuoka, Japan.

## Taxonomy

Morphological comparison with some congeners revealed that the two unidentified species of *Platygaster* are distinct species and new to science. They are described below as *P. ingeniosus* and *P. urniphila*. The two new species share typical morphological characteristics of *Platygaster* and are distinctly different from the exceptional species mentioned in the Introduction.

### Platygaster ingeniosus

Taxon classificationAnimaliaDipteraCecidomyiidae

Matsuo & Yamagishi sp. n.

http://zoobank.org/AEE14D9B-872E-446A-93C7-56270048F020

#### Etymology.

The specific name is derived from its ingenious parasitoid strategy.

**Type material.** Holotype: Female, emerged on 16 March 2011 from a gall of *Masakimyia pustulae* on *Euonymus japonicus* collected by T. Fujii from Nijoshikaka, Itoshima, Fukuoka, Japan. Paratypes: 5 females and 5 males, same data as holotype.

#### Description.

**Figure 1.**
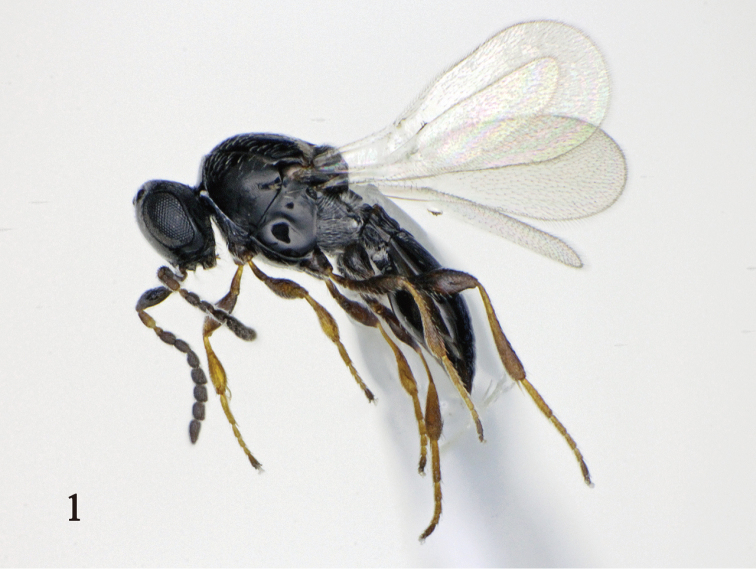
Holotype female of *Platygaster ingeniosus*.

FEMALE (Fig. 1). Body length 1.4–1.6 mm. Head, mesosoma, and metasoma black. A1 dark brown basally; A2–A4 dark brown to black; A5–A10 black. Fore wing slightly infuscate. All coxae black; all femora brown yellow to black; all tibiae brown yellow.

**Figure 2–5.**
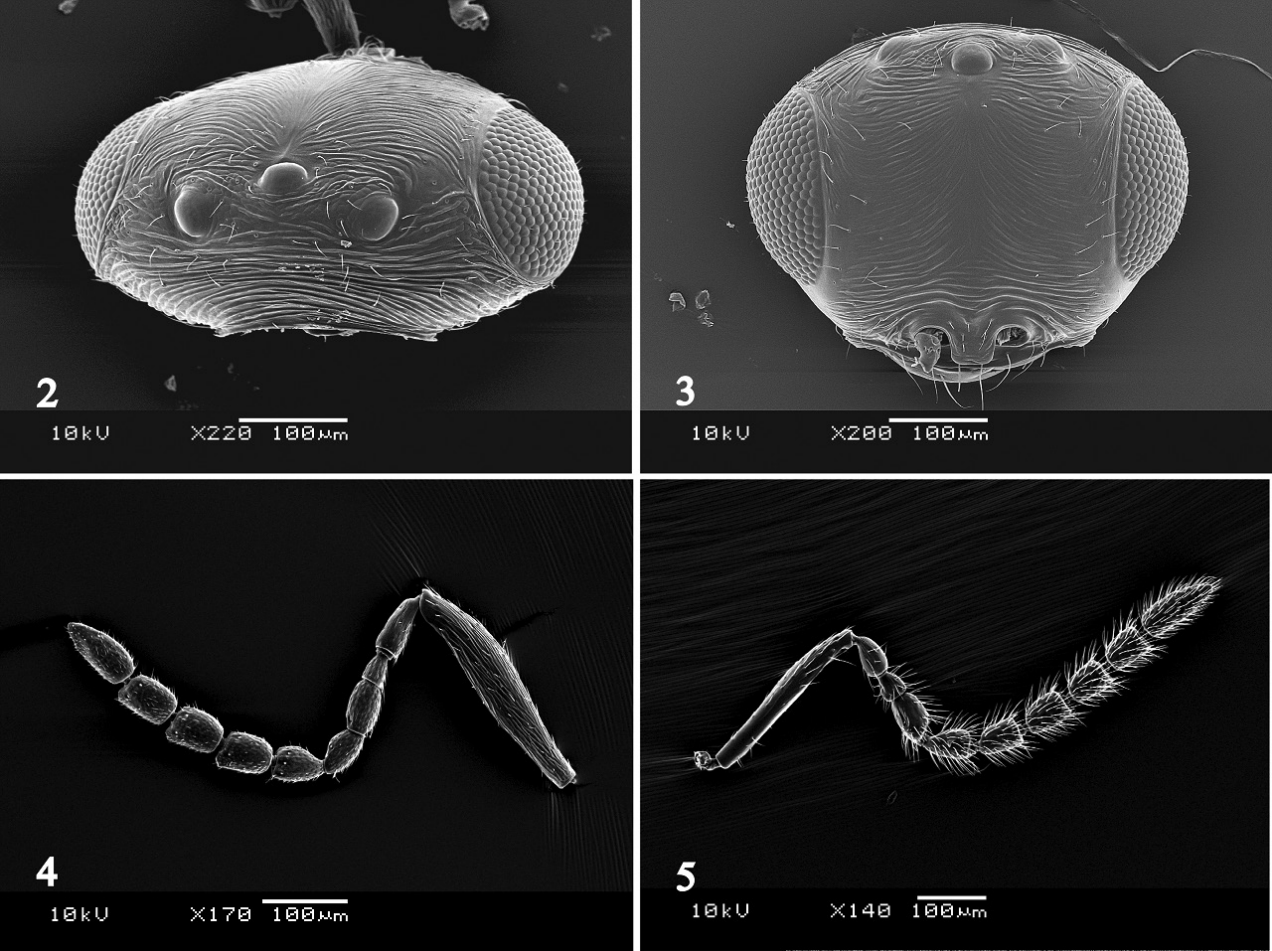
*Platygaster ingeniosus*. **2** female head, dorsal view **3** female head, frontal view **4** female antenna **5** male antenna.

Head in dorsal view, 1.9–2.1 times as wide as long, 1.1–1.2 times as wide as mesosoma; occiput transversely striate; vertex between ocelli with transverse wrinkles, with reticulation between posterior ocelli (Fig. 2); POL: OOL: LOL = 2.5: 1.3: 1.0. Head in frontal view 1.3–1.4 times as wide as high; frons with transverse wrinkles (Fig. 3); gena smooth. A1 5.5–5.6 times as long as wide, 0.7–0.8 times as long as height of head; A2 2.4–2.6 times as long as wide; A3 1.2–1.3 times as long as wide; A4–A6 1.4–1.5 times as long as wide; A7–A9 1.4–1.5 times as long as wide; A10 1.7–1.8 times as long as wide (Fig. 4).

**Figure 6–9.**
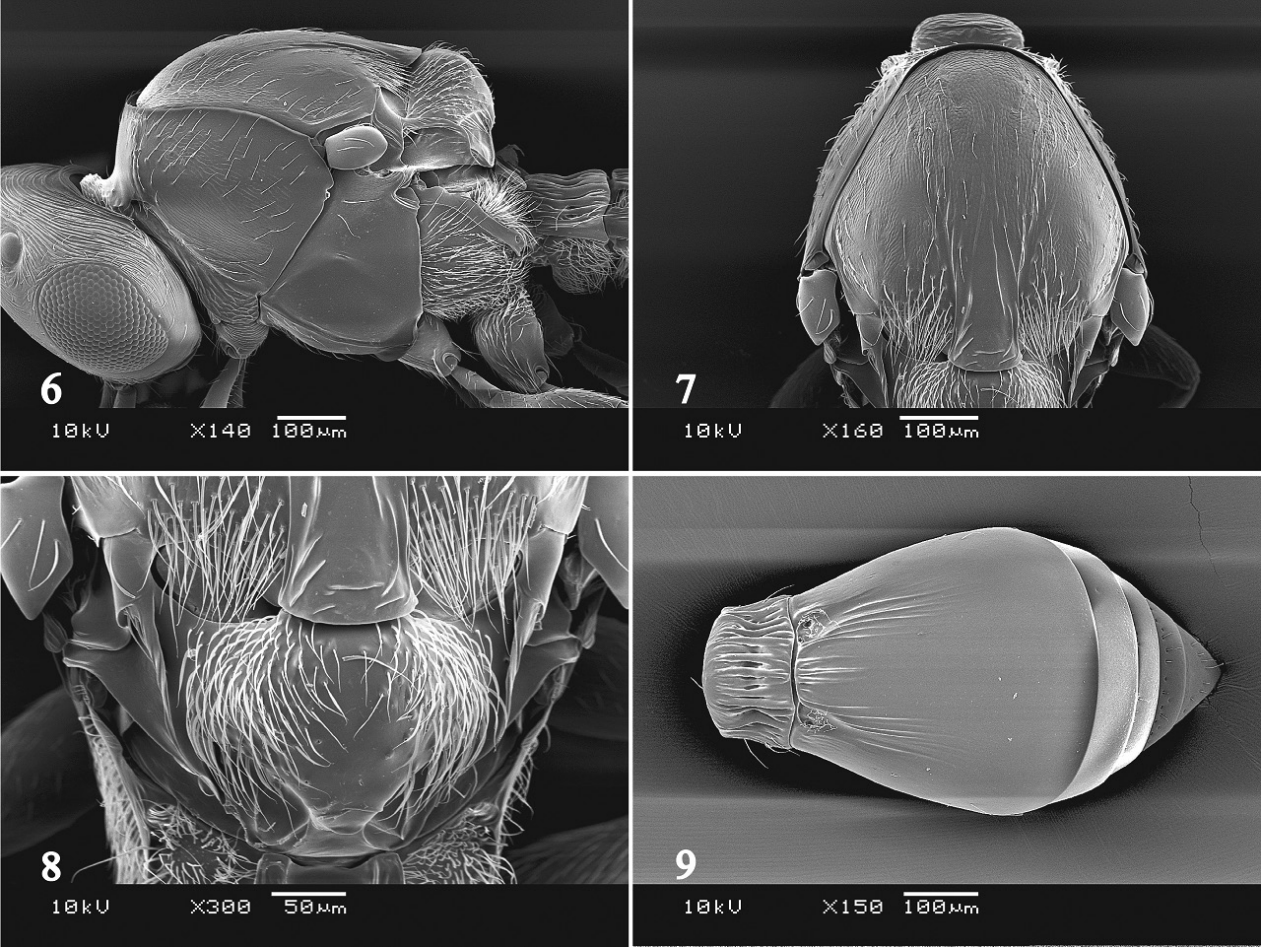
*Platygaster ingeniosus*. **6** female mesosoma, lateral view **7** female mesoscutum, dorsal view **8** female mesoscutellum, dorsal view **9** female metasoma, dorsal view.

Mesosoma 1.3–1.4 times as long as wide, 1.1–1.2 times as high as wide; sides of pronotum reticulate, smooth along upper and posterior margins (Fig. 6); mesoscutum reticulate, smooth between notauli which are indicated in posterior half (Fig. 7); posterior margin of median lobe of mesoscutum overlapping base of mesoscutellum; posterior margin of lateral mesoscutal lobes hairy; scuto-scutellar groove smooth and bare; mesoscutellum evenly convex, smooth and covered with long hairs except median glabrous area (Fig. 8); mesopleuron with two setae anteriorly, with a coriaceous area below tegula; mesopleural carina absent; mesofurcal pit present; metapleuron completely pilose; propodeal carinae widely separated, parallel. Fore wing 2.3–2.4 times as long as wide; marginal cilia approximately 0.1 times as long as width of fore wing. Hind wing approximately 4.8 times as long as wide, with two hamuli; marginal cilia approximately 0.2 times as long as width of hind wing.

Metasoma as long as head and mesosoma combined; T1 evenly crenulated, 1.8–1.9 times as wide as long, 0.2–0.3 times as long as T2; T2–T5 with a band of shallow punctation along posterior margin; T2 weakly striated in basal half, with shorter striae medially (Fig. 9); T3 with a few setae; T4 with a row of setae which is broken medially; T5 with a complete setal row; T6 with a complete setal row, smooth.

MALE. Differs from the female as follows: Body length 1.5–1.6 mm. Antenna with erect setae; A4 distinctly widened (Fig. 5). Metasoma approximately 0.8 times as long as head and mesosoma combined, obtuse at apex.

#### Differential dagnosis.

*Platygaster ingeniosus* is similar to the two Palearctic species, *P. rutilipes* Buhl and *P. yunnanensis* Buhl, because they share the following morphological characteristics: notaulus indicated in posterior half; mesopleuron with a coriaceous area below tegula; posterior margin of mesoscutum reaching base of mesoscutellum; hind wing approximately 4.8 times as long as wide. *Platygaster ingeniosus* can be distinguished from *P. rutilipes* by having the stouter fore wing that is 2.3–2.4 times as long as wide whereas *P. rutilipes* has elongated fore wing, approximately 2.8 times as long as wide. *Platygaster ingeniosus* could be distinguished from *P. yunnanensis* because sides of pronotum are finely reticulate whereas smooth in *P. yunnanensis*.

#### Biological notes.

*Platygaster ingeniosus* is an egg-larval solitary parasitoid of *Masakimyia pustulae* in Japan (Yukawa and Sunose 1976; Sunose 1983, 1984; Fujii et al. 2014). Although *Masakimyia pustulae* induces dimorphic leaf galls, thick and thin types (Sunose 1983), *Platygaster ingeniosus* can attack gall midge larvae inhabiting both types of gall (Sunose 1984; Fujii et al. 2014). Because the genus *Masakimyia* is monotypic and its tribal position in the supertribe Lasiopteridi has not been determined (Gagné and Jaschhof 2017), the possible host range of *Platygaster ingeniosus* is restricted to *Masakimyia* alone at this moment.

### Platygaster urniphila

Taxon classificationAnimaliaDipteraCecidomyiidae

Matsuo & Yamagishi sp. n.

http://zoobank.org/0F1C3380-1D61-4754-9B97-9C0EA75F16CD

#### Etymology.

The specific name, *urniphila*, is derived from the jar-shaped gall of *Rhopalomyia longitubifex*.

#### Type material.

Holotype: Female, emerged on 2–4 April 2014 from a globular-jar shaped gall of *Rhopalomyia longitubifex* on Artemisia indica var. maximowiczii collected by K. Matsuo and Y. Matsuguma on 9 November 2013 from Chojabaru, Kokonoe, Oita, Japan. Paratypes: 1 female and 1 male, same data as holotype. 3 females, emerged on 31 March 2008 from a globular-jar shaped gall of *Rhopalomyia longitubifex* on Artemisia indica var. maximowiczii collected by K. Matsuo on 8 December 2007 from Jizoubaru, Kokonoe, Oita, Japan. 2 females, emerged on 8 April 2008 from a globular-jar shaped gall of *Rhopalomyia longitubifex* on Artemisia indica var. maximowiczii collected by K. Matsuo on 2 March 2008 from Chojabaru, Kokonoe, Oita, Japan. 2 females, emerged on 13 April 2008 from a globular-jar shaped gall of *Rhopalomyia longitubifex* on Artemisia indica var. maximowiczii collected by N. Wachi on 12 April 2008 from Kuju, Taketa, Oita, Japan. 5 males, emerged in April 2016 from a globular-jar shaped gall of *Rhopalomyia longitubifex* on Artemisia indica var. maximowiczii collected by K. Matsuo and Y. Matsuo on 24 March 2016 from Tano, Kokonoe, Oita, Japan.

#### Description.

**Figure 10.**
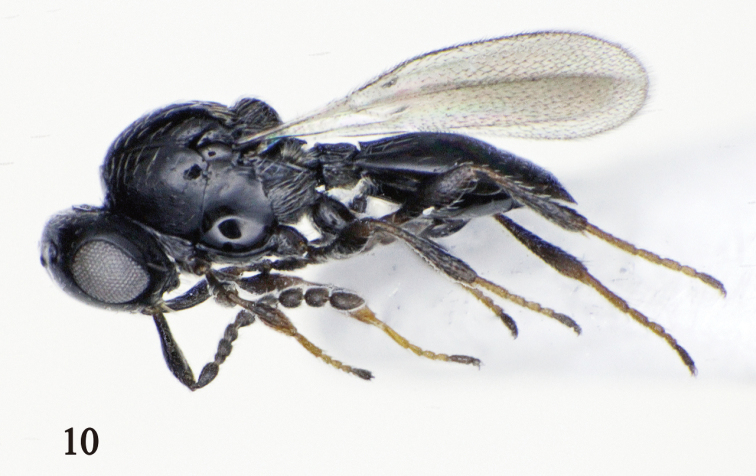
Holotype female of *Platygaster urniphila*.

FEMALE (Fig. 10). Body length 1.1–1.3 mm. Head, mesosoma, and metasoma black. A1–A2 black; A3–A10 dark brown to black. Fore wing slightly infuscate. All legs dark brown to black.

**Figure 11–14.**
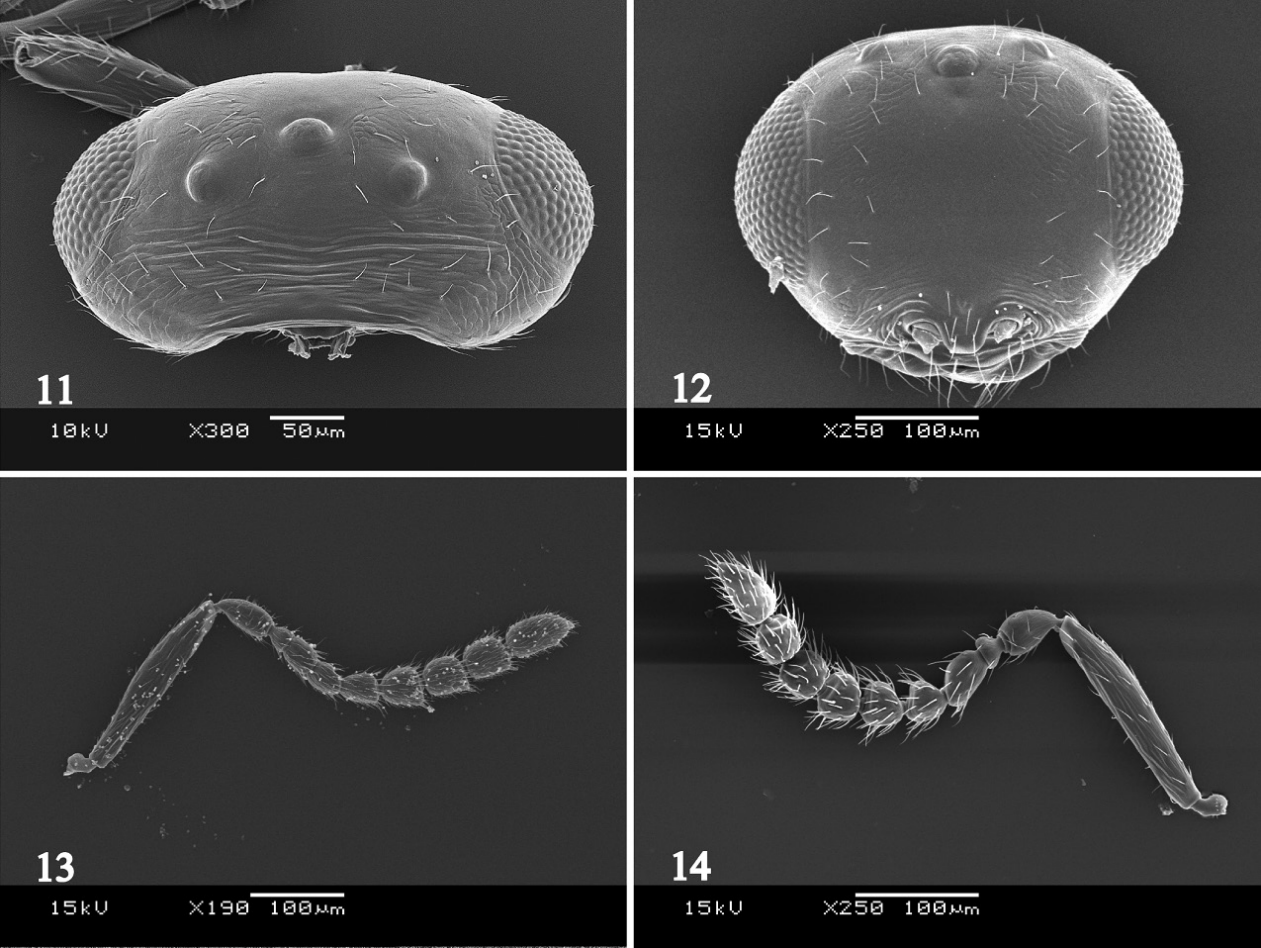
*Platygaster urniphila*. **11** female head, dorsal view **12** female head, frontal view **13** female antenna **14** male antenna. Scale bar 100 µm.

Head in dorsal view, 1.7–1.8 times as wide as long, 1.0–1.1 times as wide as mesosoma; occiput with weak transverse striations; vertex between ocelli smooth (Fig. 11); POL: OOL: LOL = 2.4: 1.0: 1.0. Head in frontal view 1.2–1.3 times as wide as high; frons smooth medially (Fig. 12), sometimes with fine striations; gena reticulate. A1 5.7–5.9 times as long as wide, 0.7–0.8 times as long as height of head; A2 1.5–1.6 times as long as wide; A3 quadrate; A4–A6 subquadrate, 1.1–1.2 times as long as wide; A7–A9 1.1–1.3 times as long as wide; A10 1.4–1.6 times as long as wide (Fig. 13).

**Figure 15–18.**
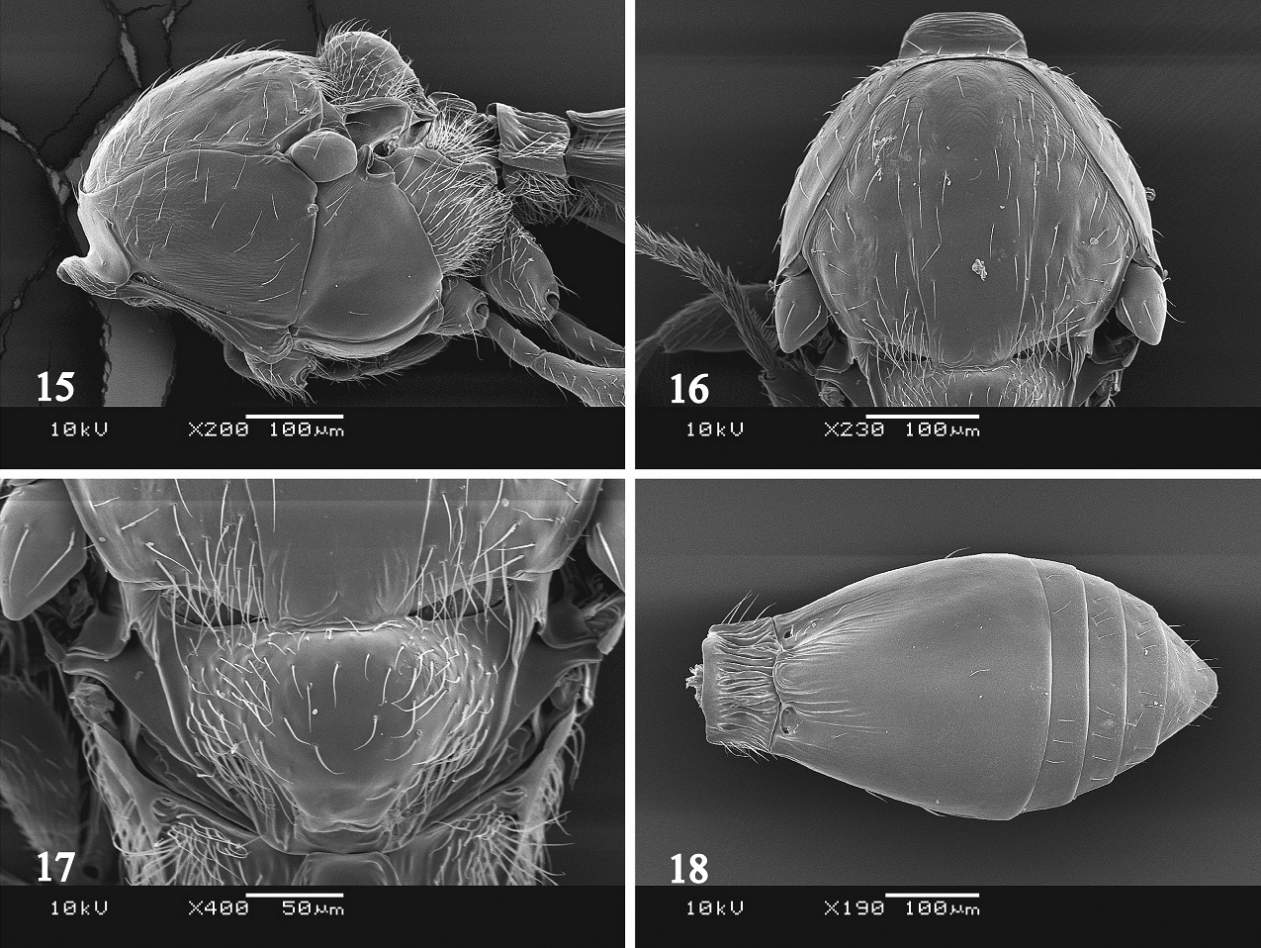
*Platygaster urniphila*. **15** female mesosoma, lateral view **16** female mesoscutum, dorsal view **17** female mesoscutellum, dorsal view **18** female metasoma, dorsal view.

Mesosoma as high as wide, 1.3–1.4 times as long as wide; sides of pronotum broadly smooth which is sometimes with extremely fine striae, smooth along posterior margin (Fig. 15); mesoscutum smooth in posterior half; notauli indicated in posterior half (Fig. 16); posterior margin of median lobe of mesoscutum not reaching base of mesoscutellum, with numerous long setae laterally; scuto-scutellar groove smooth and bare; mesoscutellum distinctly convex, smooth and covered with long hairs except median glabrous area (Fig. 17); mesopleuron with two setae anteriorly, with a coriaceous area below tegula; mesopleural carina absent; mesofurcal pit present; metapleuron pilose, sparse in dorsal one-third; propodeal carinae widely separated, parallel. Fore wing approximately 2.4 times as long as wide; marginal cilia approximately 0.1 times as long as width of fore wing. Hind wing approximately 5.3 times as long as wide, with two hamuli; marginal cilia approximately 0.2 times as long as width of hind wing.

Metasoma as long as head and mesosoma combined; T1 evenly crenulated, 1.7–1.8 times as wide as long, 0.2–0.3 times as long as T2; anterior margin of T2 weakly produced and overlapped T1; T2 weakly striated in basal half, with shorter striae medially (Fig. 18); T2–T5 with a band of shallow punctuation along posterior margin; T3–T5 with a row of setae which is broken medially; T6 with a setal row which is sometimes sparse medially, smooth.

MALE. Differs from the female as follows: Body length 1.1 mm. Antenna with erect setae; A4 distinctly widened; A5–A9 quadrate (Fig. 14). Metasoma approximately 0.8 times as long as head and mesosoma combined, obtuse at apex.

#### Differential diagnosis.

*Platygaster urniphila* can be distinguished from *P. urnicola* Yamagishi, a Japanese species, based on the following characteristics: mesopleuron with a few setae anteriorly (glabrous in *P. urnicola*); posterior margin of median lobe of mesoscutum not reaching base of mesoscutellum (reaching base of mesoscutellum in *P. urnicola*). *Platygaster gifuensis* was described based on a single male from Japan, from which *P. urniphila* can be distinguished by having A5–A9 quadrate (approximately 1.5 times as long as wide in *P. gifuensis*). *Platygaster urniphila* is quite similar to *P. sublongicornis* Buhl because they share the following morphological characteristics: vertex between ocelli smooth; frons smooth medially; mesopleuron with a few setae anteriorly, with a coriaceous area below tegula; mesoscutellum distinctly convex; T2 weakly striated in basal half, with shorter striae medially. However, *Platygaster urniphila* can be distinguished from *P. sublongicornis* based on the following characters: A4–A5 subquadrate (distinctly elongate in *P. sublongicornis*); OOL as long as LOL (1.6 times as long as LOL in *P. sublongicornis*); sides of pronotum smooth along posterior margin (smooth along anterior and posterior margins in *P. sublongicornis*); hind wing approximately 5.3 times as long as wide (4.5 times in *P. sublongicornis*).

#### Biological notes.

*Platygaster urniphila* is an egg-larval gregarious parasitoid of *Rhopalomyia longitubifex* that induces axillary bud galls on Artemisia indica var. maximowiczii in Japan (Yukawa and Masuda 1996; Ganaha et al. 2007). Gall polymorphism has been found in *R. longitubifex*: long jar-shaped, jar-shaped, and globular jar-shaped (see figures 1–5 of Ganaha et al. 2007). At present, *P. urniphila* has been reared only from globular jar-shaped galls. Various sorts of galls induced by *Rhopalomyia* spp. have been found on *Artemisia* spp. (e.g. Yukawa and Masuda 1996; Yukawa 2014; Gagné and Jaschhof 2017), but *P. urniphila* has been reared only from galls of *Rhopalomyia longitubifex* on A. indica var. maximowiczii in Japan. Future intensive studies are needed to confirm the host range of *P. urniphila*.

Leiby and Hill (1924) noted that *Platygaster vernalis*, a polyembryonic species, occasionally laid male and female eggs into a single host egg. Thus, *P. vernalis* has both polyembryonic and gregarious reproductive strategies. Our rearing experiments indicated that *P. urniphila* is a gregarious parasitoid because males and females were reared from a single host larva (Table 1). To confirm polyembryonic reproduction by *P. urniphila*, we need histological survey or MIG-seq analysis (Suyama and Matsuki 2015) that discriminate individuals originated from clonal division and sexual reproduction.

**Table 1.** Reproduction by *Platygaster urniphila*: the number of adults emerged from a single larva of *Rhopalomyia longitubifex*.

**Collecting date**	**Locality**	**Number of broods examined**	**Number of *P. tubiphila* emerged per larva (Mean ± SE)**		
			**Female**	**Male**	**Total (Female + Male)**
8 December 2007	Jizoubaru, Kokonoe, Oita, Japan	1	29	0	29
2 March 2008	Chojabaru, Kokonoe, Oita, Japan	1	7	0	7
12 April 2008	Kuju, Taketa, Oita, Japan	1	8	0	8
9 November 2013	Chojabaru, Kokonoe, Oita, Japan	1	18	1	19
24 March 2016	Tano, Kokonoe, Oita, Japan	11	16.6 ± 1.4	2.7 ± 0.4	19.4 ± 1.6
18 March 2017	Machida, Kokonoe, Oita, Japan	23	11.4 ± 0.9	1.3 ± 0.3	12.7 ± 0.9

## Supplementary Material

XML Treatment for Platygaster ingeniosus

XML Treatment for Platygaster urniphila

## Supplementary materials

Supplementary material 1 A list of papers that were used for morphological comparison Data type: bibliographic records This is an open access article distributed under the terms of the Creative Commons Attribution License (CC BY 4.0), which permits unrestricted use, distribution, and reproduction in any medium, provided the original author and source are credited.Kazunori Matsuo, Tomohisa Fujii, Makoto Tokuda, Tomoko Ganaha–Kikumura, Junichi Yukawa, Kenzou Yamagishi
